# Reverse Visually Guided Reaching in Patients with Parkinson's Disease

**DOI:** 10.1155/2022/8132923

**Published:** 2022-03-28

**Authors:** Pauline Gaprielian, Stephen H. Scott, Ron Levy

**Affiliations:** ^1^Centre for Neuroscience Studies, Queen's University, Kingston, Ontario, Canada; ^2^Department of Biomedical and Molecular Sciences, Queen's University, Kingston K7L 3N6, Ontario, Canada; ^3^Department of Medicine, Queen's University, Kingston, Ontario, Canada; ^4^Department of Surgery, Kingston General Hospital, Queen's University, Kingston, Ontario, Canada

## Abstract

In addition to motor symptoms such as difficulty in movement initiation and bradykinesia, patients with Parkinson's disease (PD) display nonmotor executive cognitive dysfunction with deficits in inhibitory control. Preoperative psychological assessments are used to screen for impulsivity that may be worsened by deep brain stimulation (DBS) of the subthalamic nucleus (STN). However, it is unclear whether anti-Parkinson's therapy, such as dopamine replacement therapy (DRT) or DBS, which has beneficial effects on motor function, adversely affects inhibitory control or its domains. The detrimental effects of STN-DBS are more apparent when tasks test the inhibition of habitual prepotent responses or involve complex cognitive loads. Our goal was to use a reverse visually guided reaching (RVGR) task, a hand-based version of the antisaccade task, to simultaneously measure motor performance and response inhibition in subjects with PD. We recruited 55 healthy control subjects, 26 PD subjects receiving treatment with DRTs, and 7 PD subjects receiving treatment with STN-DBS and DRTs. In the RVGR task, a cursor moved opposite to the subject's hand movement. This was compared to visually guided reaching (VGR) where the cursor moved in the same direction as the subject's hand movement. Reaction time, mean speed, and direction errors (in RVGR) were assessed. Reaction times were longer, and mean speeds were slower during RVGR compared to VGR in all three groups but worse in untreated subjects with PD. Treatment with DRTs, DBS, or DBS + DRT improved the reaction time and speed on the RVGR task to a greater extent than VGR. Additionally, DBS or DBS + DRT demonstrated an increase in direction errors, which was correlated with decreased reaction time. These results show that the RVGR task quantifies the benefit of STN-DBS on bradykinesia and the concomitant reduction of proactive inhibitory control. The RVGR task has the potential to be used to rapidly screen for preoperative deficits in inhibitory control and to titrate STN-DBS, to maximize the therapeutic benefits on movement, and minimize impaired inhibitory control.

## 1. Introduction

Parkinson's disease (PD) is a progressive neurodegenerative disorder associated with a loss of dopamine within the substantia nigra pars compacta. The loss of dopamine has been linked to disturbances of movement, behavior, and cognition [1–3]. PD is characterized by motor symptoms such as bradykinesia, rigidity, rest tremor, and postural instability [4]. Bradykinesia is defined as the slowness and amplitude reduction of a performed movement [5–7] and is readily quantified in the clinic using rapid alternating movements such as finger tapping, hand movements, pronation-supination movements, toe tapping, and foot tapping [8]. Bradykinesia is worse during complex movements such as sequential or simultaneous movements [9–11]. Akinesia, the reduction in spontaneous or associated movements and an inability to initiate movements, may be related to deficits in movement preparation (programming) and execution [6, 12–14]. Preparatory processes can be quantified by measuring reaction times in simple reaction tasks (same predefined response during each trial) and choice reaction tasks (response selected from two or more unpredictable alternatives) [14]. Simple reaction times are slower in PD [15] but deficits in choice reaction times are variable and may be dependent on predictive cues [16] and experimental design [15].

Patients with PD exhibit problems with executive cognitive functioning, specifically deficits in inhibitory control [17–23]. The assessment of inhibitory dysfunction is important to screen patients with PD for impulse-control disorders [24]. PD is clinically assessed using the Unified Parkinson's Disease Rating Scale [8]; psychiatric and cognitive deficits are quantified via self-report questionnaires and are often underreported [25]. Inhibitory control is not a single executive function but rather involves different components (e.g., motor and interference (or cognitive) inhibition), and these components have proactive and reactive domains [26]. Reactive inhibitory control is related to the ability to stop an ongoing movement, while proactive inhibitory control is the ability to shape a response strategy in anticipation of known task demands a planned movement [22]. Dopamine replacement therapy may improve response inhibition deficits in patients with short disease duration, but this effect is lost in moderate-to-advanced PD [18] or is limited to dopamine overdosing in young age of onset patients [27] or patients with dyskinesias [28, 29].

The effect of deep brain stimulation (DBS) on response inhibition is controversial. Recordings of subthalamic nucleus (STN) single-units from nonhuman primates during oculomotor tasks demonstrate that STN neurons use a switch signal from the presupplementary motor area to switch between habitual to controlled processing [30]. Electrophysiological recordings of the STN in patients with PD demonstrate changes in the oscillatory activity in both proactive response inhibition and reactive response inhibition [31, 32]. Reactive inhibition measured using the hand/arm version of the stop-signal reaction time task, where a stop signal is provided after a random delay following a go signal, has shown that bilateral STN-DBS results in significantly shorter stop-signal reaction times, thereby indicating improved reactive inhibitory control [33–35] and proactive inhibitory control [36, 37], but these results are variable [38, 39]. Bilateral STN-DBS also improves the proficiency of inhibiting upper limb movements measured with the Simon task [40], but the analysis of the EMG activity suggests that STN-DBS may weaken the suppression of the erroneous muscle activity [41]. Interestingly, unilateral STN-DBS does not affect inhibitory control of upper limb movements [42, 43]. In contrast, DBS of the STN has been shown to result in increased errors on the Stroop interference task suggesting that stimulation may adversely affect the ability to suppress habitual prepotent responses [44, 45]. Random number generation is also affected by STN-DBS with stimulation resulting in higher habitual counting [46]. STN-DBS causes delays in the preparation phase of the compensatory steps in a posture perturbation task [47]. Interestingly, bilateral STN-DBS may increase the antisaccade error rate [48] in an amplitude-dependent manner [49], indicating that complex aspects of oculomotor control can be adversely affected [50]. However, a large study did not report an adverse effect of STN-DBS with an antisaccade task [51]. Bilateral DBS may also increase impulsiveness during a decision-making task in complex cognitive task [52] or under speed pressure [53].

It has been suggested that STN-DBS impairs a patient's ability to change their behavior in novel contexts [54] especially in tasks requiring patients to perform a novel movement over a prepotent habitual response. Common habitual responses are saccades and reaching movements. In the former, errors in the inhibition of habitual pro-saccades during antisaccade trials in patients with PD have been shown to be a useful marker of generalized measures of cognitive control of complex motor behaviors involving both inhibitory and task-switching abilities (when interlaced with pro-saccades) [55, 56] and may be adversely affected by DBS [48, 49, 57] or dopamine overdosing in patients with earlier disease onset and milder motor symptoms [58]. In patients with PD, the assessment of limb movement is clinically more relevant than eye movements. In this study, we use a reverse visually guided reaching (RVGR) task as an analogue of the oculomotor antisaccade task. Our goal was to develop a standardized clinical methodology to quantify the simultaneous effects of therapy on akinesia/bradykinesia and on response inhibition. The benefit of using arm movements is that we can assess how well subjects can inhibit a habitual prepotent motor response (i.e., direct reaching) in order to perform the task under a novel sensorimotor transformation. A direction error during the RVGR task would indicate the failure of proactive response inhibition. The interactive Kinarm robotic platform has previously been shown to objectively quantify bradykinesia in subjects with PD [59]. The RVGR task has been used to demonstrate impairments in inhibitory control in individuals following a transient ischemic attack [60]. The aim of this study was to use the RVGR task to simultaneously quantify motor performance and inhibitory control in subjects with PD and to determine the effects of treatment with DRTs and DBS.

## 2. Methods

### 2.1. Subject Groups

Subjects with PD were recruited from the Movement Disorders Clinic at the Kingston Health Sciences Hotel Dieu site in Kingston, Ontario, Canada. Inclusion criteria for their participation included a diagnosis of idiopathic PD, the ability to understand basic task instructions, normal or corrected vision, and no injury limiting movement of the upper extremities. Three groups were tested: (1) subjects with PD treated with DRTs (“DRT group”), (2) subjects with PD treated with STN-DBS and DRTs (“DBS + DRT group”), and (3) healthy age- and sex-matched controls. Each control subject was carefully screened by a research assistant and a data analyst to ensure no muscular, skeletal, or neurological deficits were present. Control subjects were only analyzed if their performance values that were within two standard deviations of the Kinarm control range. There were 3 age-controlled subjects who were not included (1 with musculoskeletal and 2 with performance beyond two standard deviations from normal performance). This study was approved by the Queen's University Ethics Review Board, and informed consent was received from each subject prior to their participation in the study.

Testing of medication effects in the DRT group was not randomized (i.e., the on assessment was always performed after the off assessment). Subjects from the DRT group were tested twice on the same day: first, after experiencing a minimum 12-hour overnight washout of their Parkinsonian medication (OFF state), and second, one hour after taking their routine levodopa (ON DRT state). Testing of DBS and medication effects in the DBS + DRT group was fully randomized. The DBS + DRT group was tested over two separate days. One day they also experienced a minimum 12-hour overnight washout of their Parkinsonian medication (OFF state), and on the other, they were tested without withholding their medications (ON DRT state). During each day, the tasks were performed twice. One time after the stimulators had been turned OFF for 30 minutes (OFF DBS state) and a second time with the stimulators left ON (ON DBS state). The order of DRTs and DBS withdrawal was randomized. Implantation of the DBS electrodes was performed with microelectrode recordings targeting the dorsolateral STN. Postoperative confirmation was obtained using MRI-CT reconstruction and typical beta frequency peaks recorded from the DBS contacts. Monopolar stimulation of the dorsolateral contact that resulted in the best clinical efficacy was used, and the clinical stimulation parameters were not altered. Subjects from the control group were tested once. All subjects separately performed the tasks with their dominant and nondominant hands. Handedness was determined using the Edinburgh Handedness Inventory.

### 2.2. Tasks Details

**Figure 1:**
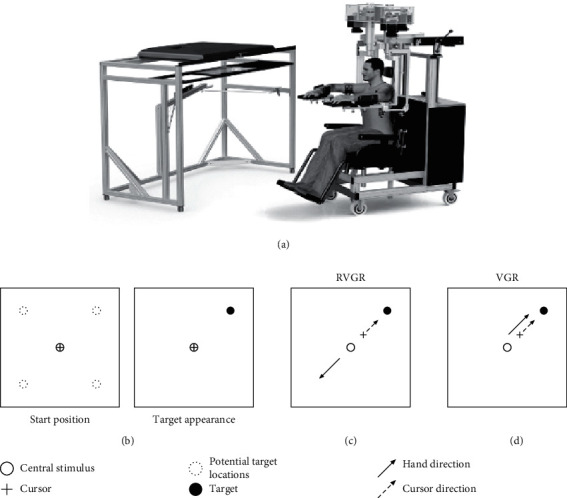
Kinarm robots and task schematics. (a) The Kinarm exoskeleton robot. (b) A schematic of the starting position and target appearance for both tasks. (c) Direction of the hand movement and cursor movement for the RVGR task. The cursor moves in the opposite direction of the hand movement. (d) Direction of the hand movement and cursor movement in the same direction for the VGR task.

Quantitative assessment was performed using the Kinarm exoskeleton robot (Kinarm, Kingston, ON). The Kinarm exoskeleton lab allows for the assessment of bimanual horizontal planar arm movements involving the elbow and shoulder joints (Figure 1(a)). Prior to the assessment, subjects were seated in an adjustable height chair and their arms placed in the exoskeleton using arm troughs where the linkage joints were aligned with the shoulder and elbow joints. A virtual reality system projected spatial targets onto the horizontal workspace as well as feedback of the hand position that was represented as a small circle. Direct visual feedback of the arms was obscured by a physical barrier. Task instructions were described prior to being performed, and verbal confirmation was received to ensure that subjects understood the task specifics and goals. Corrected vision was allowed during robotic assessment.

The task battery included two standard Kinarm tasks—RVGR and visually guided reaching (VGR). Participants performed the VGR followed by the RVGR. The difference between the two tasks is that in RVGR, the cursor moves opposite to the subject's hand direction, whereas in the VGR, the cursor was coregistered with the hand and moves with it. The VGR task is a choice reaction time and movement speed task. Here, the subjects prepare a prepotent habitual reaching movement and perform this as a go-only trial. The RVGR is similarly performed except that the subject must prepare and execute a novel reaching movement, the reverse reach. The RVGR measures the ability to inhibit a prepotent habitual motor response (direct reaching) and generate a voluntary motor response in the opposite direction. A direction error is a proactive inhibition error because the subject has difficulty in shaping their response strategy in anticipation of the known task demands. In addition, a reactive stop signal is generated from a self-cued error when subjects realize they have made a direction error.

Both tasks begin with the appearance of a central stimulus, and the subject moves the cursor to this point. In the RVGR task, the cursor originally is aligned with the hand motion, but only begins to move in the opposite direction to that of the hand after arriving at the central stimulus. After a random time interval, 1 of 4 peripheral target locations, each located 10 cm diagonal from the start target, is displayed (Figure 1(b)). In both tasks, the subjects were instructed to move the cursor quickly and accurately to the target. This movement is shown in Figures 1(c) and 1(d) for RVGR and VGR, respectively. Once the target is reached, the original start location reappears and the subject must navigate back to it. Upon reaching the central stimulus, a peripheral target is again displayed. The order of the targets was random. In the VGR task, subjects had to complete 40 trials per limb, within a trial duration of 3 seconds. In the RVGR task, subjects had to complete 48 trials per limb within a trial duration6 seconds. The intratrial interval in both tasks was 1250–1750 m·sec.

The precise 2-D path trajectories of movements were recorded. This provided a measure of trajectory length travelled from start point to the target allowing the accurate measure of movement speed. In both tasks, data regarding reaction time and movement speed for each successful trial were collected. Reaction time was calculated as the time between the appearance of a peripheral target and the onset of movement [61]. Movement speed was calculated by dividing a subject's movement time by their movement trajectory length. Movement time was quantified as the total time from the onset to the offset of movement [61]. Direction errors were calculated as the initial movement of the cursor at a distance of +/−pi/2 away from the presented peripheral target and were used to quantify proactive response inhibition. The mean reaction times, mean speeds, and number of directional errors were reported for each subject.

### 2.3. Data Analysis

The analysis protocol for the RVGR task in the Kinarm standard battery combines data from the outward movements with back movements into a single parameter [60]. In subjects with PD, it has previously been shown that reaching movements are performed faster to known target locations compared to unexpected or unknown locations [62]; therefore, we only analyzed their outward movements from the central stimulus to the peripheral target. The parameter values for mean reaction time, mean speed, and total directional errors between the dominant and nondominant hands were averaged. Reaction times and mean speed between RVGR and VGR were compared using Wilcoxon signed rank test. Comparisons between cohorts of the effect of the reverse reaching on movement performance used ANOVA on ranks of the percent change in reaction times (between VGR and RVGR), the percent change in mean speeds (between VGR and RVGR), and the number of direction errors. Multiple pairwise comparisons were performed using Dunn's method. The correlation between parameter values was determined with the Pearson product-moment correlation test. Statistical analysis was performed using Sigma Plot 11.0 (Systat Software Inc., USA), and significance was set at *P* < 0.05

## 3. Results

Data from healthy subjects were collected from 55 individuals (“control group”). A total of thirty-three subjects with Parkinson's disease were included in this study.  Table 1 shows the demographics and DRT levodopa equivalent dosages for each subject. From the recruited thirty-three, twenty-six subjects were being treated with DRTs alone (PD01-PD26, “DRT group”) and seven with a combination of DRTs and STN-DBS (PD27-PD33, “DBS + DRT group”).

### 3.1. The Effect of Reverse Reaching on Reaction Time and Mean Speed

**Figure 2:**
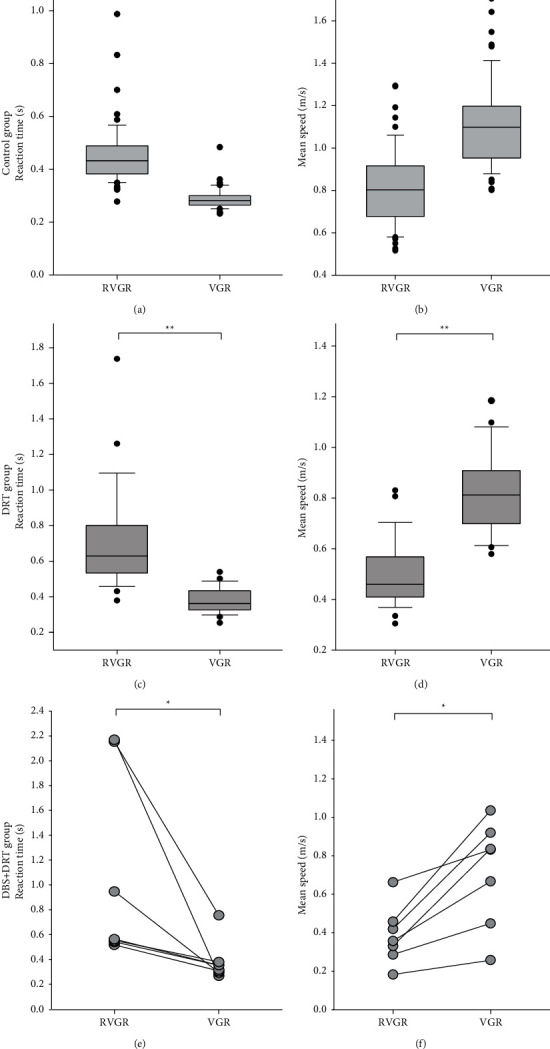
Changes in reaction time and mean speed in control subjects and subjects with PD off therapy. Mean reaction times were longer, and mean speeds were slower during RVGR compared to VGR in the control group (^*∗∗*^*P* < 0.001) (a, b), the DRT group (^*∗∗*^*P* < 0.001) (c, d), and the DBS + DRT group (^*∗*^*P* < 0.05) (e, f). Median values with 25% and 75% quartiles and outliers (marked by black dots) are shown.

Control group subjects demonstrated significant differences in reaction time and movement speed in the RVGR task compared with the VGR task. Mean reaction time during the VGR task was a median of 0.28 sec, which increased to 0.43 sec during RVGR (Wilcoxon signed rank test, *Z* = −6.45, *P* < 0.001) (Figure 2(a)). Mean speed for the VGR task was a median of 1.10 m/s, which was reduced to 0.80 m/s during the RVGR task (Wilcoxon signed rank test, *Z* = 6.393, *P* < 0.001) (Figure 2(b)).

In the OFF state, one subject from the DRT group (subject ^#^21) experienced severe akinesia using their dominant hand and could not perform the reaching movements with their dominant hand before the trial timed out. This subject was able to perform the RVGR and VGR tasks with their nondominant hand. All the other subjects in the DRT group could perform the tasks with both hands. In the DRT group during the OFF state, the mean reaction time during the VGR task was a median of 0.36 sec, which increased to 0.63 sec during RVGR (Wilcoxon signed rank test, *Z* = −4.46, *P* < 0.001) (Figure 2(c)). Mean speed for the VGR task was a median of 0.81 m/s, which was reduced to 0.46 m/s during the RVGR task (Wilcoxon signed rank test, *Z* = 4.46, *P* < 0.001) (Figure 2(d)).

In the DBS + DRT group during the OFF state, the mean reaction time during the VGR task was a median of 0.32 sec, which increased to 0.56 sec during RVGR (Wilcoxon signed rank test, *Z* = −2.37, *P*=0.016) (Figure 2(e)). Mean speed for the VGR task was a median of 0.83 m/s, which was reduced to 0.36 m/s during the RVGR task (Wilcoxon signed rank test, *Z* = 2.37, *P*=0.016) (Figure 2(f)).

The effect of the reverse reaching on reaction time in all three cohorts was compared. Reaction time increased between VGR to RVGR by 51% in the control group, 75% in the DRT group, and 82% in the DBS + DRT group (*P*=0.003). There was also a statistical difference in the means speeds between VGR to RVGR with reverse reaching (Kruskal–Wallis one-way analysis of variance on ranks, H = 24.09 with 2 degrees of freedom, *P* < 0.001). Mean speed decreased by 24% in the control group, 41% in the DRT group, and 46% in the DBS + DRT group. Pairwise comparison showed that there was a difference between control subjects and both groups of subjects with PD (Dunn's method, *Q* = 2.9 and *Q* = 4.4, *P* < 0.05) but not between the PD groups.

### 3.2. DRT Effects on Reaction Time and Mean Speed in DRT Group

**Figure 3:**
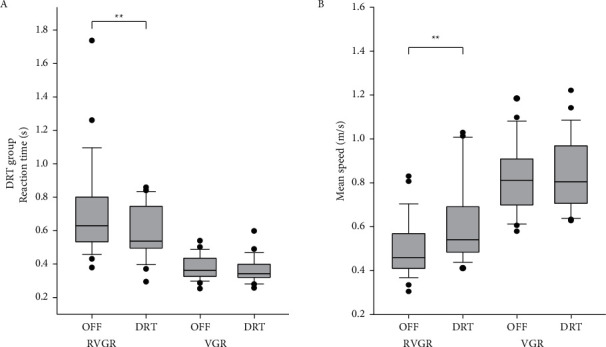
DRT group reaction time and mean speed changes with DRT administration in the DRT group. (a) DRT administration resulted in faster reaction times during RVGR (^*∗∗*^*P* < 0.001). (b) DRT administration resulted in higher RVGR mean speeds (^*∗∗*^*P* < 0.001).

All subjects were able to perform the RVGR and VGR tasks with both arms after the administration of DRTs. After DRT administration, the reaction time decreased by 12% in the RVGR task (Wilcoxon signed rank test, *Z* = −3.365, *P* < 0.001) but was not changed in the VGR task (Wilcoxon signed rank test, *Z* = −1.66, *P*=0.10) (Figure 3(a)). The change in the reaction time following DRT was greater in the RVGR task compared to the VGR task (Wilcoxon signed rank test, *Z* = 2.248, *P* < 0.05).

RGVR mean speed increased by 14% with DRT (Wilcoxon signed rank test, *Z* = 4.457, *P* < 0.001), but no change was observed for VGR (Wilcoxon signed rank test, *Z* = 0.60, *P*=0.56) (Figure 3(b)). The percent change in mean speed due the DRT administration was significantly greater in RVGR compared to VGR (Wilcoxon signed rank test, *Z* = −4.05, *P* < 0.001).

### 3.3. Treatment Effects on Reaction Time and Mean Speed in DBS + DRT Group

**Figure 4:**
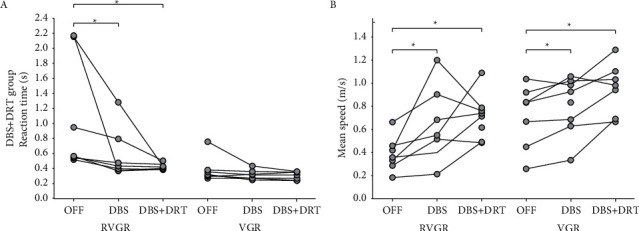
DBS + DRT group performance. (a) Reaction times were reduced by DBS and by DBS + DRT during RVGR (^*∗*^*P* < 0.05) but not during VGR. (b) Mean speeds were increased by DBS and by DBS + DRT during RVGR (^*∗*^*P* < 0.05) and during VGR (^*∗*^*P* < 0.05).

Figure 4(a) shows the change in each subject's reaction time values with DBS or DBS + DRT in the RVGR task. There was a significant treatment effect (Friedman RM ANOVA on ranks, chi-square = 11.14 with 2 degrees of freedom, *P* < 0.001) with DBS therapy reducing reaction time by 23% (Student–Newman–Keuls method, *Q* = 4.8, *P* < 0.05) and DBS + DRT reducing reaction time by 31% (Student–Newman–Keuls method, *Q* = 4.54, *P* < 0.05). Mean speeds were significantly increased with DBS or DBS + DRT in the RVGR task (Friedman RM ANOVA on ranks, chi-square = 10.57 with 2 degrees of freedom, *P*=0.003) (Figure 4(b)). Mean speed increased by 36% with DBS (Student–Newman–Keuls Method, *Q* = 5.35, *P* < 0.05) and 100% with DBS + DRT (Student–Newman–Keuls method, *Q* = 4.16, *P* < 0.05).

Unlike the changes observed during the RVGR task, there was no effect of DBS or DBS + DRT on reaction time during VGR (Friedman RM ANOVA on ranks, chi-square = 6.00 with 2 degrees of freedom, *P*=0.051)(Figure 4(a)). VGR mean speed was increased (Friedman RM ANOVA on ranks, chi-square =10.29 (2), *P*=0.004) by a median of 11% with DBS (Student–Newman–Keuls, *q* = 3.21, *P* < 0.05) and by a median of 32% with DBS + DRT (pairwise comparisons Student–Newman–Keuls method, *q* = 3. 4.54, *P* < 0.05) (Figure 4(b)).

### 3.4. Direction Errors

**Figure 5:**
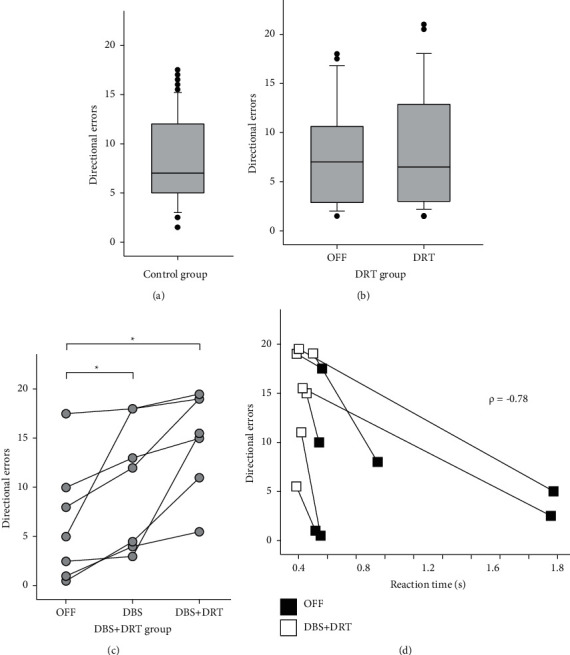
Direction errors during RVGR. (a). Control group. (b) DRT group. There was no effect of DRT on directional errors. (c) DBS + DRT group. Total directional errors were increased with DBS and DBS + DRT (^*∗*^*P* < 0.05). (d). The change in directional errors versus the change in reaction time for off therapy and DBS + DRT. The changes in directional errors with DBS + DRT in were correlated to the changes in mean reaction time (*P*=0.04).

There was no difference in the number of direction errors produced during RVGR between cohorts off treatment (Kruskal–Wallis one-way analysis of variance on ranks, *H* = 2.27 with 2 degrees of freedom, *P*=0.32). In the control group, 15% of the RVGR trials (a medium of 7 out of 48 trials) had direction errors. During the OFF state, the DRT group had direction errors in 15% of the RVGR trials, while DBS + DRT group subjects had 10% of the RVGR trials with direction errors (Figure 5(a)–5(c)). There was no significant correlation between reaction time and direction errors in the control group (Pearson product-moment correlation, *P*=0.135), DRT group (Pearson product-moment correlation, *P*=0.483), or the DBS + DRT group (Pearson product-moment correlation, *P*=0.561).

DRT did not alter the proportion of direction errors in the DRT group (Wilcoxon signed rank test, *Z* = 1.62, *P*=0.11) (Figure 5(b)). In the DBS + DRT group, both DBS and DBS + DRT significantly increased the proportion of direction errors made (Friedman RM ANOVA on ranks, chi-square = 14.000 with 2 degrees of freedom, *P* < 0.001). The proportion of direction errors was 25% with DBS (Student–Newman–Keuls, *q* = 3.74, *P* < 0.05) and 32% with DBS + DRT (Student–Newman–Keuls, *q* = 5.29, *P* < 0.05) (Figure 5(c)).  Figure 5(d) shows the relationship between the reaction time and direction error. There was a significant correlation between the reduction in reaction time and the increase in direction errors following DRT and DBS therapy (Pearson product-moment correlation, correlation coefficient = −0.776, *P*=0.04).

## 4. Discussion

This study demonstrates that the RVGR task can simultaneously measure treatment effects on motor performance and response inhibition in subjects with PD. Both cohorts of subjects with PD experienced greater decrements in performance when they had to perform a reverse reach in the RVGR task compared to the control group. The main finding is that the use of a visuo-motor transformation in a reaching movement better detects the effect of DRT, DBS, and DBS + DRT in comparison with habitual reaching movements. This is consistent with previous findings showing that the addition of a cognitive load to a motor task has greater sensitivity in differentiating mild and moderate disability in PD [63]. Patients with PD may also have a slower movement speed when external cues are reduced [64] or to unanticipated external visual targets [62]. Our results may also be related to the deficits in PD of executing internally guided movements that require planning prior to a movement [65]. Subjects with PD display increased difficulty performing a visuo-motor transformation to an unanticipated target due to the increased difficulty of the task [66], which may be attributed to cognitive executive dysfunction [67] and impaired speed-accuracy trade-offs with increasing task difficulty [68].

We consistently observed a greater effect of DRT and DBS + DRT on the RVGR task compared with the VGR task. This is consistent with previous work showing that relatively simple tasks such as button presses are less affected by DRT [69]. Complex movements such as sequential or simultaneous more improved by levodopa than simple movements [70]. These findings suggest that the RVGR is more sensitive to the effect of therapies to treat PD. The greatest change was with concurrent STN-DBS and DRT treatment consistent with previous studies examining finger flexion/extension movements [71]. STN-DBS has also been shown to significantly improve movement time of sequential movement but with decreased accuracy [72]. It has been proposed that STN-DBS upregulates the gain of all components of movement rather than improving focused control [73].

The STN is thought to play a major role in inhibitory control and impulse-control disorders [74]. Deficits in executive control and response inhibition in PD may be due to the inability to stop habitual responses and can be observed using the Stroop test [20, 75] or with increased choice complexity during a choice reaction task [76]. Impairments in inhibiting ongoing responses can be shown with stop-signal tasks [19]. Countermanding tasks using no-stop trials compared with unexpected stop-signal trials are useful in demonstrating deficits in both reactive and proactive inhibitory control in patients with PD [22, 23]. In the present study, both groups of untreated PD subjects and the control group made a similar number of direction errors. However, DBS and DBS + DRT significantly affected direction errors in comparison with subjects treated with DRT alone. This suggests that STN-DBS may induce deficits in proactive response inhibition and reduce cognitive flexibility to override habitual responses with novel actions. In addition, the faster reaction times in DBS + DRT subjects with a higher proportion of direction errors suggest an increase in impulsivity and release of a habitual prepotent response. Interestingly, the direction error trajectories were corrected accordingly indicating that reactive response inhibition can be used after a self-cued error to correct a motor plan.

In patients with PD, increased impulsivity is related to impaired motor and behavioral inhibitory control [28] and may worsen following STN-DBS in spite of motor benefit [54, 77, 78]. The detrimental effects of STN-DBS on executive cognitive function in patients with PD have been shown using a variety of tasks such as the go/no go paradigm [79–81], Stroop task [45, 54, 82], and antisaccade task [48–50, 83]. It has been hypothesized that these effects are related to DBS of the ventral STN leading to impairments in automating responses in tasks with increased cognitive loads [45, 80, 81]. Assessment of the effects of STN-DBS on oculomotor control in PD using the antisaccade task has also shown improved motor performance coupled with impaired cognitive control with stimulation [48–50, 83]. Specifically, bilateral stimulation of the STN produced lower saccade latencies (RTs), increased fixation during the preparatory period, increased amplitudes, and increased error rates in the antisaccade task [48–50, 83]. Goelz et al. also reported an association between the number of saccades that were made during the fixation/preparatory period and the onset of errors [83]. These findings indicate that while bilateral STN-DBS may benefit the motor aspects of oculomotor control, it can negatively impact the cognitive aspects via a disruption in the cortico-basal ganglia circuit.

A variety of tasks have been used to measure inhibitory dysfunction in PD. The go/no go, antisaccade, and Stroop task paradigms have been employed for the assessment of inhibitory control in subjects with PD [20, 79, 84, 85]. The results from these tasks have highlighted the inability of PD patients to inhibit automatic responses towards a presented stimulus [56, 79, 86]. These tasks demonstrate that the loss of inhibition exhibited by PD patients may worsen with treatment with STN-DBS [44, 48, 79]. However, one of the most commonly used tests to assess inhibitory control, the stop-signal task, shows that bilateral STN-DBS results in significantly shorter stop-signal reaction times, suggesting improved reactive inhibitory control [33–35] and proactive inhibitory control [36, 37]. These assessment methodologies have provided reliable information regarding the assessment of PD and the effects of STN-DBS. However, the drawbacks of these methodologies are the duration of their assessment protocols and their inability to provide information regarding how a loss of inhibitory control is related to bradykinesia, which is commonly used to titrate DRT or DBS therapy. Specifically, the testing protocol for the go/no go paradigm is between 180–1000 trials in length [87]. The antisaccade protocol requires subjects to perform 60 pro-saccade trials followed by 120 antisaccade trials and 60 more pro-saccade [88]. The Stroop test can take up to 120 minutes to complete [89]. Unlike the mentioned assessment methodologies, the RVGR task is 48 trials in length and takes only 3 minutes to complete per limb. Furthermore, the RVGR task can simultaneously provide information regarding bradykinesia and response inhibition. This is similar to the stop-signal task, which relates inhibitory control to bradykinesia by evaluating the context effect [90].

Neuropsychological evaluation of DBS potential candidates is essential to the selection process and surgical success [91, 92]. However, there is no standardized protocol for neuropsychological assessment. Instead, there is an extensive list of neuropsychological domains that are assessed using a number of commonly used tests [93]. The drawbacks of the clinically recommended testing batteries available are that they are subjective and time-consuming, and may not be specific to PD impairments. Additionally, the identification of optimal stimulation settings depends on a step-by-step process in which a clinician increases stimulation voltages in increments of 0.2–0.5 V until therapeutic benefit is seen in the absence of adverse side effects [91, 94, 95]. This process is limited by a clinician's experience, as they have to use subjective measures of response inhibition to quantify therapeutic benefits and side effects. Furthermore, clinicians cannot accurately quantify the effects of stimulation voltage on cognitive impairment, which is considered the primary side effect of treatment with DBS. Finally, the step-by-step process is often time-consuming and exhausting to patients, which may confound the results [91]. The RVGR task offers a helpful alternative to the clinically available scales by providing a rapid and objective assessment of the therapeutic benefits and negative side effects of STN-DBS.

There are methodological limitations of this study. The lack of randomization in the DRT group is a limitation of the study. There may be learning effects in the DRT group that contributed to their improved performance when ON medication. It has been shown that Kinarm tasks with higher cognitive burdens, such as RVGR, may be more vulnerable by learning effects [96]. Although each subject performed 40 trials per limb, this is actually 4 times the number of repetitions that are used to quantify movement in the MDS-UPDRS and showed consistent effects even in the DBS + DRT cohort with 7 patients. Lastly, the block design is not necessarily a limitation. The subjects all had a priori knowledge of the task requested but still make significant direction errors in the RVGR task. In addition, the block design is similar to how clinicians measure UPDRS bradykinesia where 10 repetitions of a movement are used to determine a score. Differences between cohorts include the progression of visuospatial deficits related to disease duration (DBS + DRT group mean 17.5 years, DRT group mean 5.5 years) [97]. Another difference between the cohorts is the microlesioning effect of the DBS electrode. The brain penetration effects of DBS electrodes in the STN can improve motor function for up to 6 months following penetration [98]. A limitation to clinical implementation of this methodology is the cost of the Kinarm assessment technology ($200–300K Canadian). However, the utility of employing this system is the standardization of the testing apparatus and data analysis in clinical trials and in the clinical environment. Another advantage of this technology is that it can be used to quantify a broad range of sensory, motor, and cognitive processes in a variety of disorders such as postoperative delirium [99], stroke [100], transient ischemic attack [60], amyotrophic lateral sclerosis [101], and neurological impairments associated with non-neurological diseases [102]. The versatility of the Kinarm assessment technology could justify the cost. Future studies will involve the assessment of RVGR in the operating room and clinic to determine the clinical utility of this task in DBS electrode implantation and adjustments to therapeutic stimulation.

## 5. Conclusions

The current study demonstrates that the RVGR task can be used to simultaneously quantify the motor effects and response inhibition in subjects with PD. This task has the potential to be used as an assessment tool in the screening of appropriate DBS candidates, in the identification of optimal stimulation settings, and for quantifying the adverse effects of STN-DBS on impulsivity.

## Figures and Tables

**Table 1:** Characteristics of subjects with PD.

Participant code	Age (years)	Sex	Disease duration (years)	Years with DBS	PD medications	Levodopa equivalent dosages (mg/day)
PD01	48	M	0.5	N/A	Levodopa	600
PD02	55	M	6	N/A	Levodopa	600
PD03	71	M	5	N/A	Levodopa, stalevo	1332
PD04	67	M	5	N/A	Levodopa, rasagiline, pramipexole	825
PD05	70	M	15	N/A	Levoodpa, stalevo	1365
PD06	67	M	11	N/A	Levodopa, amantadine	600
PD07	55	M	3	N/A	Levodopa	550
PD08	72	M	13	N/A	Levodopa	350
PD09	57	M	4	N/A	Levodopa	600
PD10	69	M	6	N/A	Levodopa	875
PD11	72	M	8	N/A	Levodopa, pramipexole	550
PD12	70	F	5	N/A	Levodopa, stalevo	858
PD13	53	F	2	N/A	Levodopa	1100
PD14	61	F	3	N/A	Levodopa	300
PD15	73	M	3	N/A	Levodopa, pramipexole	900
PD16	75	M	8	N/A	Levodopa, pramipexole	950
PD17	69	F	5	N/A	Levodopa, pramipexole	700
PD18	72	M	5	N/A	Levodopa, pramipexole	1300
PD19	68	M	5	N/A	Levodopa, pramipexole	1125
PD20	75	M	3	N/A	Levodopa	950
PD21	58	M	5	N/A	Levodopa	600
PD22	54	F	4	N/A	Levodopa	450
PD23	75	F	8	N/A	Levodopa	600
PD24	47	M	3	N/A	Levodopa	450
PD25	64	M	6	N/A	Levodopa, rotigotine	1120
PD26	71	F	2	N/A	Levodopa	400
PD27	63	M	20	5	Levodopa, rotigotine	1170
PD28	56	M	19	3	Levodopa	2475
PD29	75	F	21	4	Levodopa	900
PD30	46	M	14	5	Levodopa, ropinirole	920
PD31	63	M	10	0.25	Levodopa, pramipexole, amantadine	476
PD32	77	F	16	7	Levodopa, pramipexole, rasageline	475
PD33	63	M	23	5	Levodopa	500

## Data Availability

The datasets used and analyzed during the current study are available from the corresponding author upon reasonable request.
